# Multiplex ligation-dependent probe amplification (MLPA) enhances the molecular diagnosis of aniridia and related disorders

**Published:** 2008-05-07

**Authors:** Egbert J.W. Redeker, Annette S.H. de Visser, Arthur A.B. Bergen, Marcel M.A.M. Mannens

**Affiliations:** 1 Department of Clinical Genetics, Academic Medical Centre, University of Amsterdam, Meibergdreef 15, 1105 AZ Amsterdam, The Netherlands; 2 Department of Clinical and Molecular Ophthalmogenetics, The Netherlands Institute for Neuroscience, an institute of the Royal Netherlands Academy of Arts and Sciences (KNAW), Amsterdam, The Netherlands

## Abstract

Mutations in the *PAX6* gene have been implicated in aniridia, a congenital malformation of the eye with severe hypoplasia of the iris. However, not all aniridia cases can be explained by mutations in the *PAX6* gene. The purpose of this study was to enhance the molecular diagnosis of aniridia using multiplex ligation-dependent probe amplification (MLPA). Total genomic DNA was isolated from peripheral blood of 70 unrelated probands affected with aniridia. Polymerase chain reaction (PCR) was performed followed by automated bidirectional sequencing. Additionally, MLPA was performed. We identified 24 different point mutations in the *PAX6* gene in 34 patients after sequencing. In eight additional patients, we identified a deletion of one or more exons of the *PAX6* gene or in the 3′ regulatory region of the *PAX6* gene using MLPA. This work demonstrates the necessity to screen for larger deletions in the region of the *PAX6* gene in addition to the sequencing of exons in the *PAX6* gene. The mutation detection rate will increase from 49% to 60%. This shows that MLPA substantially enhances the molecular diagnosis of aniridia.

## Introduction

Aniridia (AN2; OMIM 106210) is a genetic disorder, which is characterized by iris hypoplasia as well as several other ocular features such as cataract, glaucoma, corneal opacity, and nystagmus. The aniridia gene was mapped to chromosome 11p13, and no other locus besides chromosome 11p13 was convincingly implicated in aniridia [1]. Heterozygous mutations in the *paired box gene 6* (*PAX6*; OMIM 607108), located on human chromosome 11p13, were previously implicated in familial and sporadic aniridia. The functional relationship between the gene and the disease was further enhanced by the finding that *PAX6* mutations cause the small eye (sey) phenotype in mice [2]. *PAX6* is clearly the major if not only gene responsible for the disease. A frequently updated summary of *PAX6* mutations, which are associated with aniridia and related disorders, is documented at the *PAX6* allelic variation database [3]. *PAX6* is highly conserved, and missense mutations can also cause dysfunction of the protein [4]. In isolated aniridia cases, published *PAX6* alterations comprise mainly point mutations [1]. The majority of these mutations result in premature termination of the PAX6 protein. A proportion of aniridia cases are associated with the WAGR (Wilms tumor, aniridia, genitourinary abnormalities, mental retardation) syndrome. WAGR is a contiguous gene syndrome arising from deletions of chromosome 11p13, which encompass at least both the *PAX6* and *WT1* genes. In contrast, only a few isolated aniridia patients with a gross deletion or a cytogenetic translocation in the *PAX6* region have been reported in the literature [5-10]. Gross deletions may be underreported due to the fact that these are not always detected by FISH (fluorescent in situ hybridization) and that these are not detected by the common mutation detection methods such as SSCP, DHPLC, or sequencing.

We performed a mutation analysis for *PAX6* in 70 unrelated patients with aniridia. By scanning the 14 exons of *PAX6*, 24 different point mutations were identified in 34 patients. In addition, we detected gross deletions in another eight patients using multiplex ligation-dependent probe amplification (MLPA).

## Methods

### Patients

The patient group consisted of 70 patients who were referred to our laboratory with the clinical diagnosis of isolated aniridia. The study was performed according to a protocol approved by the local ethics committee.

**Table 1:** List of primers used to perform amplification and sequencing of the 14 *PAX6* exons.

**Exon**	**Forward primer**	**Reverse primer**
1	AGGGAACCGTGGCTCGGC	GGGTGAGGGAAGTGGCTGC
2	TTATCTCTCACTCTCCAGCC	GGAGACCTGTCTGAATATTGC
3	TCAGAGAGCCCATCGACGTAT	CTGTTTGTGGGTTTTGAGCC
4	AGTTCAGGCCTACCTGATGC	GTCGCGAGTCCCTGTGTC
5	CTCCCTCATCTTCCTCTTCC	GGGGTCCATAATTAGCATCG
6–7	GGGCTACAAATGTAATTTTAAGAAA	AGAGAGGGTGGGAGGAGGTA
8	GAGCTGAGATGGGTGACTG	GAGAGTAGGGGACAGGCAAA
9	AGACTACACCAGGCCCCTTT	TGAAGATGTGGCATTTACTTTGA
10	GGAACCAGTTTGATGCACAG	ACTCTGTACAAGCACCTCTGTCTC
11	GGGCTCGACGTAGACACAGT	GGAAACTGAGGGCAAGAGAA
12	CGGGCTCTGACTCTCACTCT	GCCACTCCTCACTTCTCTGG
13	GCTGTGGCTGTGTGATGTGT	AGGAGATTCTGTTTGGGTA
14	TCCATGTCTGTTTCTCAAAGG	TCAACTGTTGTGTCCCCATAG

Sequences of primers used to perform amplification and sequencing of the 14 *PAX6* exons.

**Figure 1:**
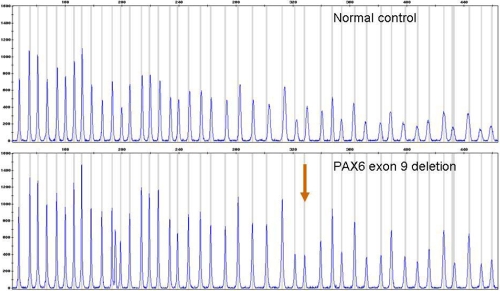
Detection of *PAX6* exon 9 deletion by MLPA. Electropherograms are from a normal control and from the patient with an exon 9 deletion. The deletion is apparent by a ~50% reduction in peak area of the *PAX6* exon 9 specific probe (red arrow).

### DNA analysis

Genomic DNA was isolated from peripheral blood. All 14 exons of the *PAX6* gene were amplified and sequenced bidirectionally on an ABI PRISM 377 sequencer (Applied Biosystems, Foster City, CA) using standard technology and the primers described in Table 1. The mutation nomenclature is according to the recommendations of the Human Genome Variation Society [11]. cDNA nucleotide numbering (reference sequence NM_001604.3 from GenBank) starts with +1 at the A of the ATG translation initiation codon. Probes for MLPA analysis (SALSA MLPA Kits P097 and P219) were purchased from MRC Holland (Amsterdam, the Netherlands). MLPA was performed according to the manufacturer’s instructions. In summary, 100 ng DNA was denatured and hybridized overnight at 60 °C with SALSA probe mix. After treating the samples with ligase 65 for 15 min at 54 °C, polymerase chain reaction (PCR) amplification was performed with the specific SALSA FAM PCR primers. Electrophoresis of PCR products was performed using an ABI PRISM 310 (Applied Biosystems). Data analysis was performed by exporting the peak areas to a Microsoft Excel file. Sample-related and peak-related differences in PCR and electrophoresis efficiency were corrected by first calculating the peak area relative to the sum of peak areas per sample and subsequently expressing each normalized peak area relative to the mean of that peak across samples. To detect deviating peaks, each normalized peak was divided by the mean of that peak in the control samples. Peak areas outside the range 0.7–1.3 times the control peak area were considered abnormal with those below 0.7 representing deletions and those above 1.3 representing duplications. All normal samples run in one assay showed standard deviations of less than 10% of the mean. Several control samples were included in each MLPA experiment. Each result was confirmed by two independent tests.

### Long range polymerase chain reaction

For confirmation of the *PAX6* exon 9 deletion, PCR was performed with the following primer combination: 5′-GGA CTT CGG TGC CAG GGC AAC CTA-3′ (forward) located in exon 8 and 5′-TGG CTG CTA GTC TTT CTC GGG CAA ACA-3′ (reverse) located in exon 10.

### Fluorescent in situ hybridazation

FISH analysis was performed according a standard protocol using the FISH probes, FAT5 (*PAX6*), p60 (D11S324), and B2.1 (WT1) as described by Crolla et al. [7].

## Results

We performed a mutation analysis for *PAX6* in 70 unrelated patients with isolated aniridia. By scanning the 14 exons of the *PAX6* gene, 24 different point mutations were identified in 34 patients (Table 2). We identified the following types of mutations: a frameshift mutation in nine patients; a splice-site mutation in seven patients; a nonsense mutation in 11 patients; in three patients a no-stop change; and in four patients a missense mutation.

**Table 2:** Point mutations in the *PAX6* gene.

**Patient**	**DNA change**	**Consequence**	**Reference**
1 and 53	c.-52+1G>A	Splice defect	[15]
6 and 74	c.301delG	p.Glu101LysfsX22	Database*
9	c.489T>G	p.Tyr163X	New
11, 12	c.399+5G>A	Splice defect	New
16	c.181_189delinsCA	p.Tyr61GlnfsX15	New
19	c.34G>C	p.Gly12Arg	New
20	c.406C>T	p.Gln136X	[16]
21	c.-52+5G>C	Splice defect	New
22, 48, 75	c.1268A>T	p.X423Leu	[17]
23, 29, 79	c.718C>T	p.Arg240X	[18]
26	c.640A>G	p.Arg214Gly	New
30, 49	c.114_121del8	p.Pro39HisfsX13	New
35	c.844_845delCC	p.Pro282X	New
36	c.112delC	p.Arg38GlyfsX15	New
37	c.117insGGCC	p.Cys40GlyfsX15	New
41, 51, 68	c.949C>T	p.Arg317X	[16]
42	c.299G>A	p.Trp100X	[19]
44	c.77G>C	p.Arg26Pro	New
58	c.365C>A	p.Ser122X	New
61	c.400–1G>C	Splice defect	New
63	c.143T>A	p.Val48Glu	New
64	c.1074+1G>A	Splice defect	[15]
66	c.265_266insC	p.Gln89ProfsX2	New
76	c.781C>T	p.Arg261X	[15]

The asterisk indicates the Human *PAX6* Allelic Variant Database.

It is known that *PAX6* missense mutations may give rise to variant phenotypes [4]. Of the four patients with missense mutations in our study, three have phenotypes within the classical aniridia spectrum. The fourth case, patient 44 with the p.Arg26Pro mutation, is a familial aniridia case with an affected mother and daughter. The daughter has ectopic pupils and marked atrophy of the iris stroma as well as congenital nystagmus and cataract. The mother has nystagmus, microcornea, coloboma of the iris with iridocorneal adhesions, some stromal atrophy, and peripheral coloboma of the choroid of one eye.

**Figure 2:**
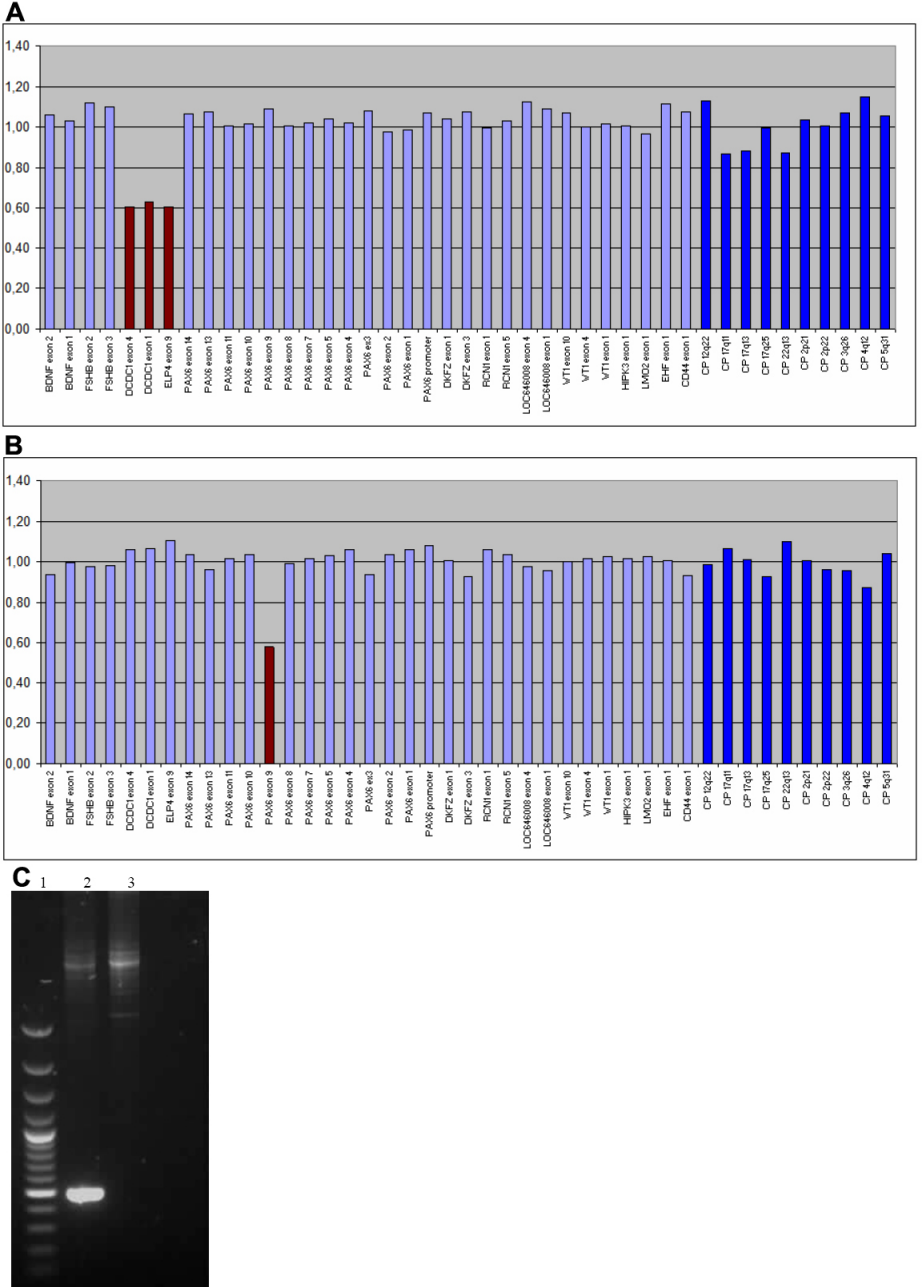
Example of normalized MLPA results. **A** and **B**: The height of the columns represents the dosage of the respective segments in the genomic DNA with two alleles (value of about one corresponds to two alleles). The light blue columns represent the 11p13 specific probes from telomere to centromere. The brown columns represent the deleted probes. The allele dosage of the deleted probes was found in the range of about 0.5, which corresponds to one allele. The dark blue columns represent the control probes. **C**: Long range PCR for *PAX6* exons 8–10 confirmed the deletion. The deleted allele is more strongly amplified than the normal allele due to preferential amplification of smaller fragments. Lane 1 is a 100 bp ladder size marker; lane 2 is the DNA of the patient; and lane 3 is a control DNA.

We examined the remaining 36 patients in which no mutation was yet identified by means of the multiplex ligation-dependent probe amplification (MLPA) method. An example MLPA result is shown in Figure 1. Using the P219 MLPA kit, a deletion was detected in eight isolated aniridia patients (Table 3). In six cases, a deletion of several contiguous exons was found; in one case, a deletion of a single exon was found; and in another case, a deletion outside the coding region of *PAX6* was identified. These first six deletions were confirmed with the MLPA P097 kit. This MLPA kit is an early version of the *PAX6* kit with probes for a limited number of *PAX6* exons. The deletion of exon 9 was confirmed by long range PCR. The last deletion is a deletion of three contiguous probes of *ELP4* and *DCDC1* (Figure 2).

**Table 3:** Gross deletions identified by MLPA kit P219 in eight isolated aniridia patients and in two WAGR patients as well as the results of the FISH analysis on these patients.

**Patient**	**Deleted PAX6 exons**	Other deleted probes*	**Confirmation**	**Deleted FISH probes**
4	exon 8–14	-	MLPA kit P097	-
5	exon 8–14	-	MLPA kit P097	-
17	exon 9	-	Long range PCR	-
45	exon 9–14	-	MLPA kit P097	-
50	exon 1–14	DCDC1 exon 4 - LOC646008 exon 4	MLPA kit P097	ND**
57	exon 1–14	ELP4 - DKFZ exon 3	MLPA kit P097	ND**
69	exon 1–14	DCDC1 exon 4- DKFZ exon 1	MLPA kit P097	-
71	-	DCDC1 exon 4 - ELP4	contiguous probes	-
WAGR1	exon 1–14	DCDC1 exon 4 – HIPK3	MLPA kit P097	FAT5, p60, B2.1
WAGR2	exon 1–14	BDNF exon 2 - CD44	MLPA kit P097	FAT5, p60, B2.1

The asterisk indicates that the first and the last deleted probes are mentioned. The double asterisk indicates that no lymphocytes were available for FISH analysis.

For validation of the MLPA method, we also analyzed the DNA of two WAGR patients with deletions that were previously detected by FISH (Table 3). One WAGR patient showed a deletion of all 11p13 probes while the other WAGR patient had a deletion of *DCDC1* exon 1 up to *HIPK3* including *PAX6* and *WT1*.

## Discussion

In this study, we showed that by using MLPA, it is possible to extend the number of confirmed aniridia cases at the molecular level. In our series of 70 isolated aniridia cases, we detected *PAX6* pathogenic sequence changes in 42 patients; 34 of whom had single nucleotide mutations (49%) and eight had deletions detected by MLPA (11%). In three of these eight cases, we found a deletion of all *PAX6* exons including the surrounding genes. In four of the eight cases, there is a partial deletion of *PAX6*. In one case, a deletion of *ELP4* and *DCDC1* was found, leaving *PAX6* itself intact. *ELP4* and *DCDC1* are located downstream of *PAX6*. Deletions in this region were described previously in aniridia patients [10,12]. This region contains 3′ regulatory elements for *PAX6* [13,14].

In spite of the multiplicity and high sensitivity of methods used, we could not give a molecular diagnosis to 40% of the patients investigated. This may be partly the result of misdiagnosis; in other cases, it might be the result of the methods used. Point mutations or deletions might occur in regions of *PAX6* that are not currently investigated such as intronic or remote promoter regions or in the 3′ regulatory region. Balanced rearrangements are not excluded either. There is no evidence to suspect that other genes besides *PAX6* are responsible for aniridia [1].

Until now, most large deletions in or near *PAX6* have been identified by FISH or Southern blot analysis. However, these techniques lack the resolution of MLPA in detecting smaller deletions or are not generally applicable to detect all kind of deletions. For our MLPA analysis, we used the P219 MLPA kit, which is an improved version of the P097 kit. Six of the eight deletions are detected by both kits. The P097 kit failed to detect two deletions as this kit lacks the relevant probes.

Young individuals with aniridia but no family history of aniridia are usually tested by high resolution cytogenetic or FISH analysis. This is done to detect a contiguous gene deletion involving 11p13 and consequently to identify individuals with an increased risk for Wilms tumor. It is now also possible to detect this type of deletion by using MLPA, which has the advantage of a higher resolution than FISH analysis and strongly reduces the time and cost of analysis.
